# Versatile Redox Chemistry Complicates Antioxidant Capacity Assessment: Flavonoids as Milieu-Dependent Antiand Pro-Oxidants

**DOI:** 10.3390/ijms140611830

**Published:** 2013-06-04

**Authors:** Vladimir Chobot, Lenka Kubicova, Gert Bachmann, Franz Hadacek

**Affiliations:** 1 Department of Molecular Systems Biology, Faculty of Life Sciences, University of Vienna, Althanstrasse 14, A-1090 Vienna, Austria; E-Mails: lenka.kubicova@univie.ac.at (L.K.); gert.bachmann@univie.ac.at (G.B.); 2 Albrecht-von-Haller Institut, Plant Biochemistry, Georg-August-Universität Göttingen, Justus-von-Liebig-Weg 11, D-37077 Göttingen, Germany; E-Mail: franz.hadacek@biologie.uni-goettingen.de

**Keywords:** hydroxyl radical, Fenton reaction, quercetin, luteolin, rutin, natural products, bioactive phytochemicals, plant stress physiology and biology, square-wave voltammetry

## Abstract

Some antioxidants have been shown to possess additional pro-oxidant effects. Diverse methodologies exist for studying redox properties of synthetic and natural chemicals. The latter are substantial components of our diet. Exploration of their contribution to life-extending or -compromising effects is mandatory. Among reactive oxygen species (ROS), hydroxyl radical (^•^OH) is the most damaging species. Due to its short half-life, the assay has to contain a specific generation system. Plants synthesize flavonoids, phenolic compounds recognized as counter-agents to coronary heart disease. Their antioxidant activities are affected by their hydroxylation patterns. Moreover, in the plant, they mainly occur as glycosides. We chose three derivatives, quercetin, luteolin, and rutin, in attempts to explore their redox chemistry in contrasting hydrogen peroxide environments. Initial addition of hydrogen peroxide in high concentration or gradual development constituted a main factor affecting their redox chemical properties, especially in case of quercetin. Our study exemplifies that a combination of a chemical assay (deoxyribose degradation) with an electrochemical method (square-wave voltammetry) provides insightful data. The ambiguity of the tested flavonoids to act either as anti- or pro-oxidant may complicate categorization, but probably contributed to their evolution as components of a successful metabolic system that benefits both producer and consumer.

## 1. Introduction

Reactive oxygen species (ROS) and antioxidants are involved in all aspects of aerobic life. All modern organisms depend on their presence as key components of physiological signaling, although, conversely, ROS can contribute to their mortality too [1,2]. In terms of ROS and other reactive species, B. Halliwell outlines the big scientific challenge for the 21st century: to understand how to stop destructive effects while preserving useful functions [3].

Numerous assays exist for characterizing the antioxidant properties of single compounds or mixtures [4–7]. Hydrogen atom transfer assays (HAT) measure the capacity of an antioxidant to scavenge free radicals by forming stable compounds (1) and consist of a synthetic free radical generator and an oxidizable molecular probe; total radical trapping antioxidant parameter (TRAP) and oxygen radical absorbance capacity (ORAC), the β-carotene or crocin bleaching assay, inhibited oxygen uptake method (IOU) and inhibition of linoleic acid or lipid autoxidation are among them.

(1) $${\text{X}}^{•}+\text{AH}\to \text{XH}+{\text{A}}^{•}$$

Electron transfer reaction-based assays (ET) monitor the kinetics of the reduction of an oxidant (probe) by the antioxidant in comparison to a standard, for example Trolox or gallic acid. The antioxidant can reduce a radical, a metal (2), or a carbonyl; total phenol assay using the Folin–Ciocalteau reagent, Trolox equivalent antioxidant capacity assay (TEAC), DPPH (2,2-diphenyl-1-picrylhydrazyl) radical scavenging capacity assay, ferric reducing/antioxidant power assay (FRAP) and Cu(II) reducing antioxidant capacity assay (CUPRAC) are classified more or less to the latter group.

(2) $${\text{M}}^{n+1}+\text{AH}\to {\text{M}}^{n}+{\text{AH}}^{•}$$

Classification to hydrogen atom transfer and electron transfer assays, however, is not always exclusive. Furthermore, assays have been introduced that measure the interference with lipid autoxidation, which is caused by free radical attack, by luminescence or electroluminescence and the formation of ethylene from α-keto-γ-methiolbutyric acid (total oxidant scavenging capacity, TOSC). The deoxyribose degradation assay was developed specifically to explore test compound or mixture capabilities to specifically scavenge hydroxyl radical (^•^OH), which is generated by reaction of an iron–ethylenediaminetetraacetic acid (EDTA) complex with hydrogen peroxide (H_2_O_2_) in presence of ascorbic acid [8,9]. Several reviewers of antioxidant capacity assays criticize *in vitro* testing for ^•^OH scavenging abilities as irrelevant arguing that the extraordinary high reactivity of ^•^OH requires the antioxidant to be present in high concentrations or that the test compound concomitantly acts by forming a complex with transition metals (iron, copper, manganese, nickel, cobalt), in which the metal (M) is a less efficient catalyst of the ^•^OH generating Fenton reaction (3) and thus is hindered to form a complex with the detection molecule 2-d-deoxyribose that causes its site-specific degradation [4–7].

(3) H2O2+Mn→O•H+OH-+Mn+1

As alternative to deoxyribose, fluorescein was proposed serving as detection molecule in the hydroxyl radical scavenging capacity assay (HOSC), which advertises the possibility to perform the assay in microtitre plates in a reaction solution volume of 300 μL [10]. Other researchers proposed inhibition of fluorescing hydroxylated terephthalate formation as alternative detection molecule of ^•^OH scavenging [11,12]. The deoxyribose degradation assay usually is performed in glass vessels. Moreover, 2-deoxyribose has less redox cycling properties than aromatic compounds which can affect ROS formation in the reaction solution [13–15].

Scavenging ROS requires a one- or two-electron transfer from the antioxidant. A recent study points to the capability of renowned antioxidants, such as cysteine, epigallocatechin gallate and glutathione, to replace ascorbic acid in a Fenton-like reaction system with albumin-sequestered copper in a wide range of pH as a pro-oxidant [16]. In the deoxyribose degradation assay, the reducing capability of ascorbic acid is used to change ferric into ferrous iron, the latter of which reduces H_2_O_2_ to ^•^OH and OH^−^. In attempts to specifically address this issue, one of us (V.C.) developed variants of the deoxyribose degradation assay that allow exploring the interactions of the test compound with the different components of the reaction mixture [17]. The exclusion of H_2_O_2_ modifies the ^•^OH-generation system in terms of speed. If, in this case, the duration of the assay is extended from 1 to 16 h, similar levels of deoxyribose degradation are detectable as with the initially highly concentrated H_2_O_2_^•^OH-generation system (4–8). This extends the detection specificity of the deoxyribose degradation assay to the development and interactions of various ROS (O_2_^•−^, H_2_O_2_, and ^•^OH), which may arise in a cascade of redox reactions (5–8) and are initiated by the reduction of molecular oxygen; in nearly all of them, iron or a comparable transition metal acts as catalyst. Exclusion of ascorbic acid then creates a reaction environment that specifically allows exploring the capability of the test compound to cause a similar pro-oxidant effect as ascorbic acid in the designated ^•^OH-generation system (4–8). Iron was added as Fe(III)−EDTA complex that was prepared separately. In the EDTA complex iron can catalyze electron transfers, but coordination with other reactants is hindered. We considered this as important for a structure–activity comparison; iron–flavonoid coordination compounds have different chemical properties.

(4) $$\text{Test compound}+{\text{Fe}}^{3+}\to \text{Test }{\text{compound}}^{•}+{\text{Fe}}^{2+}$$

(5) $${\text{O}}_{2}+{\text{Fe}}^{2+}\to {{\text{O}}_{2}}^{•-}+{\text{Fe}}^{3+}$$

(6) $${{\text{O}}_{2}}^{•-}+{\text{Fe}}^{3+}\to {\text{O}}_{2}+{\text{Fe}}^{2+}$$

(7) $${{\text{O}}_{2}}^{•-}+{{\text{O}}_{2}}^{•-}+2{\text{H}}^{+}\to {\text{O}}_{2}+{\text{H}}_{2}{\text{O}}_{2}$$

(8) H2O2+Fe2+→O•H+OH-+Fe3+

**Figure 1:**
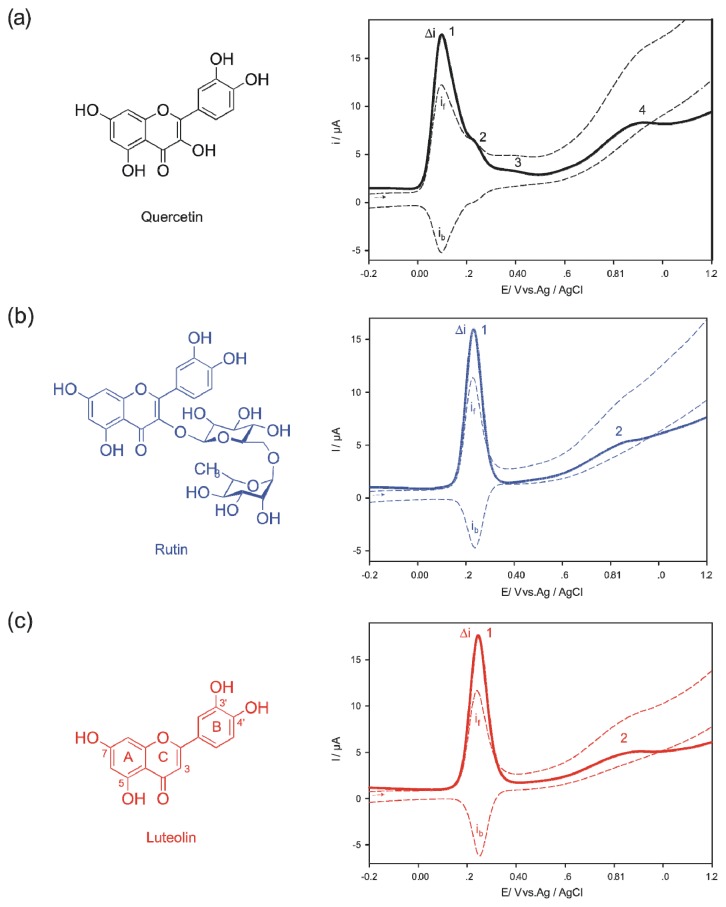
Structures and square-wave voltammograms (Δ*i* = *i*_f_ − *i*_b_, *i*_f_, forward current, *i*_b_, backward current), pH = 7.4, for details see Experimental section; (**a**) quercetin, (**b**) rutin, (**c**) luteolin.

To demonstrate the additional insights that can be obtained from applying the deoxyribose degradation assay variants, we selected three flavonoids, luteolin, quercetin, and rutin (Figure 1). Flavonoids are ubiquitously occurring phenolic compounds in the plant kingdom and well-renowned for their ability to scavenge a wide range of ROS [18]. They may be beneficial for plants that produce them by conferring to protection of the photosynthetic apparatus against oxidative stress [19,20]. Their wide-spread occurrence also causes them to be a part of our diet and an inverse correlation between phenol intake and coronary heart disease has been noted [21]. Structure–antioxidant activity relationships of flavonoids largely depend on the number of hydroxyl groups, especially on the ring B, with the highest effects correlating with catechol or pyrogallol moieties [18]. If, however, these structural characteristics are absent, the enolic 3-hydroxyl group on ring C was shown to gain importance [22,23]. The radical that arises after oxidation is stabilized by its resonance [18]. Conversely, the same flavonoids structures also are known to be pro-oxidant under certain conditions, e.g., when the availability of transition metals such as iron or copper is increased [24,25]. Not surprisingly, the *in vivo* protective efficacy of flavonoids as well as that of many other natural and synthetic antioxidants was put under question. Instead the notion arose that mild pro-oxidant effects, which stimulate antioxidant defenses, contribute more to their beneficial effects than direct ROS scavenging [3,26]. The flavonoid derivatives that were selected for this study represent variants of the previously outlined structural characteristics contributing to antioxidant activity. Luteolin lacks the enolic hydroxyl group at carbon 3 of ring C that quercetin shows and rutin is a rutinoside of quercetin (quercetin 3-*O*-α-l-rhamnopyranosyl-(1→6)-β-d-glucopyranoside) with the enolic 3-hydroxyl group masked by a disaccharide (Figure 1).

Traditionally, voltammetric methods have been used widely to study the thermodynamic aspect of redox properties [27,28]. We compare the antioxidant capacity results from the deoxyribose degradation assay variants to square-wave voltammetry (SWV), which provides peak potentials of electro-oxidation and/or -reduction as further characteristics of the test compounds in a similar chemical milieu. Square-wave voltammetry is faster, more sensitive, and requires lower amounts of electro-active species than cyclic voltammetry; both represent electrochemical methods that have been employed to study the redox properties of flavonoids in particular [29].

Here we want to show that the deoxyribose degradation assay 16 h variant without initial addition of hydrogen peroxide provides more extensive information on the potential redox chemistry of test compounds in biological systems. It fundamentally complements that of the classical deoxyribose degradation assay with initial addition of hydrogen peroxide in high concentration. Furthermore, combining both assay variants proves also highly useful for a co-interpretation with voltammetric data.

## 2. Results and Discussion

Square-wave voltammograms (SWV) of quercetin, rutin, and luteolin were measured at pH = 7.4 (Figure 1) to reflect the cytosolic pH. Quercetin (Figure 1a) showed four peaks at the potentials 0.098 V (1), 0.233 V (2), 0.388 V (3) and 0.871 V (4), respectively, in congruence with reported data; peak 1 corresponds to the oxidation of *o*-dihydroxyl groups of ring B and peak 2 to the oxidation of the hydroxyl group at position 3 of ring C [30]. The backward component of the response indicates that the products of the first and second electro-oxidation can be reduced back to quercetin. Peaks 3 and 4 are thought to be caused by the oxidation of the resorcinol moiety (*m*-dihydroxyl groups) of ring A [30]. In their case, no corresponding reversible peaks were detected in the backward response. The oxidation of the resorcinol moiety requires higher electrochemical redox potentials because its arrangement for oxidation seems to be less favorable compared to the catechol moiety. Luteolin lacks the hydroxyl group at position 3 of ring C and in rutin it is masked by disaccharide rutinose. Despite of the structural differences, the SWVs of both flavonoids are strikingly similar showing a notable peak 1 at 0.243 V and 0.233 V respectively (Figures 1b,c) [31,32]. A second but less prominent peak 2 appears at 0.834 V (luteolin) and 0.845 V (rutin). Peak 1 corresponds to the oxidation of the catechol moiety (ring B) and peak 2 to the resorcinol moiety (ring A) [31,32]. Again, only peak 1 indicated some back electro-reduction after the oxidation; however, the ratios of forward and backward current suggest the electro-reactions to be complex too. A peak that would correspond to a hydroxyl group at C ring C3 is missing accordingly.

The voltammograms suggested that complex reactions occur after analyte oxidation at the cytosolic pH. Others studies focusing on pH dependence of flavonoid electrochemistry in more detail arrived at similar conclusions [30–32]. Basically, both the elimination and masking of the C ring C3 hydroxyl group resulted in an anodic shift of the redox potential of the first maximum corroborating that rutin and luteolin possess less reductive power than quercetin.

**Figure 2:**
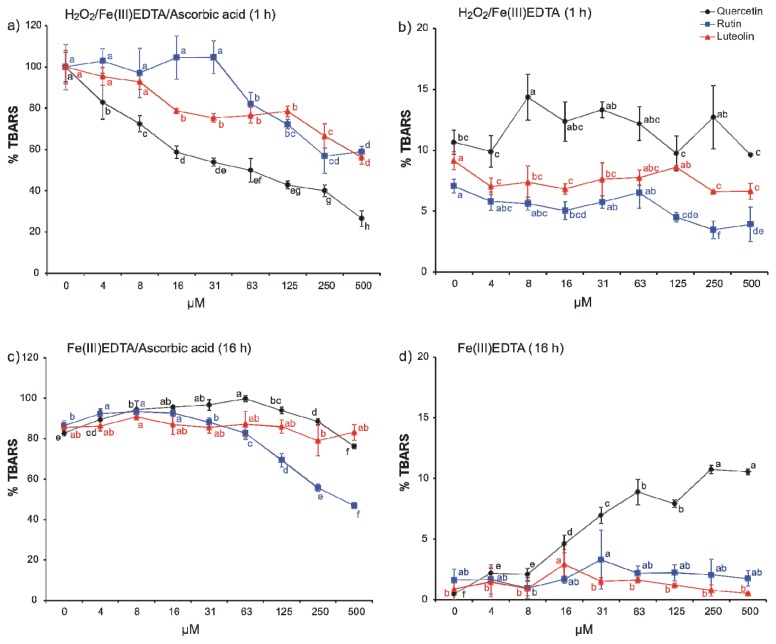
Thiobarbituric acid reactive species (TBARS) formation in (**a**) H_2_O_2_/Fe(III)EDTA/ascorbic acid (1 h incubation), (**b**) H_2_O_2_/Fe(III)EDTA (1 h incubation), (**c**) Fe(III)EDTA/ascorbic acid (16 h incubation), and (**d**) Fe(III)EDTA (16 h incubation) variants of the deoxyribose degradation assay (100% = TBARS of the control reaction mixture of the classical variant; H_2_O_2_/Fe(III)EDTA/ascorbic acid). Error bars indicate standard deviation of three replicates; letters indicate different levels of significance (95% Duncan); EDTA, ethylenediaminetetraacetic acid.

In efforts to explore the redox chemical reactions specifically with ^•^OH, the three flavonoids were subjected to various variants of the deoxyribose degradation assay [8,9,17] (Figure 2). In the standard variant, the H_2_O_2_/Fe(III)EDTA/ascorbic acid reaction mixture, quercetin proved as the most efficient scavenger of the generated ^•^OH whereas luteolin and rutin were less efficient and more similar to each other in accordance with square-wave voltammetry carried out at the same pH (Figure 2a). Correlation of redox potentials and antioxidant capacity has been pointed out by previous studies, e.g., for cyclic voltammetry and FRAP [33] and cyclic voltammetry and TEAC [34]. In terms of an activity ranking of quercetin, luteolin, and rutin, other studies, such as protection against single-strand DNA breaks [35], lipid peroxidation [36], and scavenging of the DPPH free radical [37] draw a similar picture. Quercetin has a more negative peak potential than ascorbic acid—its first peak potential against the Ag/AgCl electrode is 0.212 V—it failed, however, to substitute ascorbic acid in the reaction mixture (Figure 2b). This might have a kinetic reason.

The elimination of H_2_O_2_ from the reaction mixture has dramatic consequences on ROS availability because any H_2_O_2_ that would be available for the ^•^OH generating Fenton reaction depends on diffusion of molecular oxygen into the reaction solution and its consequent reduction [17]. As a consequence, the scoring interval was extended to 16 h. At this time point, the formation of ^•^OH from H_2_O_2_ progressed to more than 80% of the standard reaction mixture to which aqueous H_2_O_2_ was added in the beginning (1 mM H_2_O_2_ final concentration). In presence of ascorbic acid (Figure 2c), the antioxidant capacity of the flavonoids changes. Quercetin was pro-oxidant in most of the tested concentrations. Only the highest concentration, 500 μM, reduced the TBARS level to that of the control. Luteolin showed no effect, but rutin a notable antioxidant one in higher concentrations. The higher pro-oxidant capacity of quercetin was confirmed in the variant without addition of ascorbic acid (Figure 2d), in which both luteolin and rutin were inactive.

Compared to the 1 h classical variant, the 16 h variant deoxyribose degradation assay variant provides more detailed but also contrasting results on the antioxidant capacity of the three tested flavonoids. Most striking are the higher efficacy of rutin and the inefficiency of luteolin and near-to-inefficiency of quercetin to act as antioxidants in the H_2_O_2_ exclusion variant. The simultaneous assaying of a test compound in variants of the deoxyribose degradation assay, when H_2_O_2_ is either added or not added to the reaction mixture at the beginning of the assay, allows—despite being still affected by the shortcomings of an *in vitro* assay—to obtain a glimpse into the versatile and complex chemistry which flavonoids may enter *in vivo*. In plants, flavonoids usually occur as glycosides in the vacuole; the general idea is that glycosylation confers to their stability and also better solubility in aqueous solutions [38]. Interestingly, sugars, especially disaccharides have been pointed out to be able to act as antioxidants similarly as phenols, such as flavonoids [39]; similar reports also exist for sugar alcohols [40]. In the vacuole, ^•^OH may arise from H_2_O_2_ that is either formed from tonoplast-resident NADPH oxidases or diffuses through the tonoplast with or without the help of aquaporins. Attack on oligosaccharides, such as fructans, and phenols may lead to the formation of phenol and sugar radicals that either form sugar–phenol compounds, repolymerized oligosaccharides or polymerized phenols [41]. Rutin might also arise in such a scenario—rutinose is not known to occur as free sugar in nature [42] and fungal enzymes have been suggested to be involved in its biosynthesis [43]. Only the 16 h variant of the deoxyribose degradations assay reveals that rutin, in comparison to quercetin, might be a more efficient antioxidant when it is present during the entire Haber–Weiss reaction. The interaction complexity of hydroxyl radicals with low molecular weight metabolites *in vivo* represents a challenge for developing *in vitro* assays. So far, only modifications of the detection molecule in a deoxyribose degradation assay-like reaction mixture have been suggested, for example fluorescein in the HOSC estimation [10] or hydroxylated terephthalate [11], but H_2_O_2_ was added directly always into the reaction mixture at the beginning in comparatively high concentration that was required to provide quick ^•^OH formation. The herein presented results point out (1) that the speed of H_2_O_2_ generation has a substantial effect on the antioxidant capacity; and (2) a more complex ^•^OH generation system reveals a more complex and versatile redox chemistry. At the first glance, the results prove as less helpful for antioxidant capacity categorization. A careful examination, however, points out that the 16 h variant provides results that facilitate more detailed insights into the complex redox chemistry of the tested compounds, which is exemplified here by the antioxidant flavonoids. Electrochemical experiments at the same pH are highly useful to confirm redox cycling properties of certain function groups, which is required for pro-oxidant activity. They provide useful additional information but, if applied singly, fail to reflect the complex redox chemistry of the compound to a lesser extent than if used in combination with an antioxidant capacity assay. For the latter, our study points to the fact that attempts to simplify and optimize assays in terms of high throughput may affect result quality negatively.

## 3. Experimental Section

### 3.1. Chemicals

Hydrogen peroxide and 2-deoxy-d-ribose were obtained from Fluka (Buchs, Switzerland). All other chemicals used were purchased from Sigma-Aldrich (Schnelldorf, Germany). Water had Milli-Q quality.

### 3.2. Square-Wave Voltammetry

Voltammetric curves were recorded in a three-electrode system, μAutolab PGSTAT type III (EcoChemie Inc., Utrecht, The Netherlands). The working electrode was a glassy carbon electrode of 3 mm diameter, an Ag/AgCl (saturated KCl) electrode was used as reference, and platinum wire as a counter electrode. The glassy carbon electrode was washed first with methanol, then with water, and polished by aluminum oxide powder (0.3 μm of grain size) before every measurement. The effective scan rate of the voltammetry was 105 mV s^−1^, step potential was 5 mV, modulation amplitude was 25 mV, and frequency 20 Hz. The scan potential was from −0.250 to +1.200 V. The flavonoids were dissolved in degassed methanol at a concentration of 1 mM. The sample for the analysis was prepared by mixing 1 mL of this methanol solution with 9 mL of the degassed buffer (0.1 M phosphate buffer pH 7.4). The low flavonoid concentrations and presence of small amount of the organic solvent decreased the adsorption of the tested substances on the electrode surface. The electrolytes were degassed by argon for 10 min and measurements were carried out under argon atmosphere at a room temperature. Ascorbic acid was measured in a comparative fashion.

### 3.3. Deoxyribose Degradation Assay Variants

The deoxyribose degradation assay and the various variants follow published procedures [8,9,17]. Flavonoids were dissolved in aqueous KH_2_PO_4_/KOH buffer solution (30 mM, pH 7.4) and diluted serially (4–500 μM); to 125 μL of this solution, 25 μL of a 10.4 mM 2-deoxy-d-ribose solution in the same buffer system and 50 μL of Fe(III)EDTA solution (50 μM) were added. The complex of Fe(III) with EDTA was prepared separately; the 104 μM EDTA solution in the buffer was premixed with the aqueous 100 μM FeCl_3_ solution (1:1 *v*/*v*). Further, 25 μL 10.0 mM aqueous solution of H_2_O_2_ and 25 μL of 1.0 mM ascorbic acid in the buffer were added to start the Fenton reaction in the H_2_O_2_/Fe(III)EDTA/ascorbic acid reaction mixture. In the other deoxyribose degradation assays systems, H_2_O_2_ or ascorbic acid was replaced by the same volume of water or buffer, respectively. Thiobarbituric acid reactive species (TBARS) were determined photometrically at 532 nm after reaction with thiobarbituric acid and subsequent extraction of the pink pigment with 1-butanol. The H_2_O_2_/Fe(III)EDTA/ascorbic acid reaction mixture served as positive control and represented 100% TBARS detection in all variants and also served as comparative standard for each experiment. Blanks contained the full reaction mixtures except for 2-deoxy-d-ribose and were determined in each experiment. Experiments were performed in triplicates. The temperature during incubation was 27 °C. Variants containing H_2_O_2_ were evaluated after 1 hour; variants without H_2_O_2_ were evaluated after 16 h incubation. All tested compounds were explored for possible interactions with the TBARS detection procedure [44,45].

### 3.4. Statistical Analysis

Statgraphics Centurion XVI (Statistical Graphics Corp., Rockville, MD, USA) was used to perform analyses of variance (ANOVA) employing 95% Duncan’s multiple range *post hoc* test.

## 4. Conclusions

The herein presented results demonstrate that electrochemical studies of peak potentials at a physiological pH together with a variant of the deoxyribose degradation assay that monitors the whole Haber–Weiss than only the Fenton reaction alone draw a complex and probably much more realistic picture of ^•^OH interaction dynamics with low molecular weight metabolites. The unpredictability that is caused by this versatile redox chemistry may render categorizing efforts for antioxidant capacity more difficult. Conversely, this characteristic may contribute fundamentally to the success of the system as a whole, of which plant flavonoids are only a part. Low molecular weight metabolites present attractive targets to study the complex chemistry of life; large biomolecules such as proteins contain similar functional groups but in higher numbers, which substantially complicates interpretation.
